# Molecular approach to a patient’s tailored diagnosis of the oral allergy syndrome

**DOI:** 10.1186/s13601-020-00329-8

**Published:** 2020-06-17

**Authors:** Claudia Alessandri, Rosetta Ferrara, Maria Livia Bernardi, Danila Zennaro, Lisa Tuppo, Ivana Giangrieco, Teresa Ricciardi, Maurizio Tamburrini, Maria Antonietta Ciardiello, Adriano Mari

**Affiliations:** 1 Associated Centers for Molecular Allergology (CAAM), Rome, Italy; 2 Allergy Data Laboratories (ADL), Latina, Italy; 3 Institute of Biosciences and BioResources (IBBR), CNR, Naples, Italy

**Keywords:** Oral allergy syndrome, Pollen-food allergy syndrome, Food allergy, Oral cavity, Allergenic molecules, Isoallergens, Allergen isoforms, Class 1 food allergy, Class 2 food allergy

## Abstract

Oral allergy syndrome (OAS) is one of the most common IgE-mediated allergic reactions. It is characterized by a number of symptoms induced by the exposure of the oral and pharyngeal mucosa to allergenic proteins belonging to class 1 or to class 2 food allergens. OAS occurring when patients sensitized to pollens are exposed to some fresh plant foods has been called pollen food allergy syndrome (PFAS). In the wake of PFAS, several different associations of allergenic sources have been progressively proposed and called syndromes. Molecular allergology has shown that these associations are based on IgE co-recognition taking place between homologous allergens present in different allergenic sources. In addition, the molecular approach reveals that some allergens involved in OAS are also responsible for systemic reactions, as in the case of some food Bet v 1-related proteins, lipid transfer proteins and gibberellin regulated proteins. Therefore, in the presence of a convincing history of OAS, it becomes crucial to perform a patient’s tailored molecule-based diagnosis in order to identify the individual IgE sensitization profile. This information allows the prediction of possible cross-reactions with homologous molecules contained in other sources. In addition, it allows the assessment of the risk of developing more severe symptoms on the basis of the features of the allergenic proteins to which the patient is sensitized. In this context, we aimed to provide an overview of the features of relevant plant allergenic molecules and their involvement in the clinical onset of OAS. The value of a personalized molecule-based approach to OAS diagnosis is also analyzed and discussed.

## Background

The term oral allergy syndrome (OAS) describes the rapid onset of symptoms induced by food allergens on the oral and pharyngeal mucosa. These symptoms include itching and/or angioedema of the lips, tongue, palate, ears and throat, accompanied by stinging pain. In a subgroup of patients red patches or short-lasting blisters of the oral mucosa might occur. Commonly, these symptoms gradually resolve in one hour but in a few cases they might increase in severity up to anaphylactic reactions [1].

In 1942 Tuft and Blumstein gave the first description of this syndrome associating it with birch pollinosis and with a hypersensitivity to fruits and vegetables [2]. In 1987 Amlot and colleagues were the first to name these symptoms “oral allergy syndrome” describing the oral clinical manifestations induced by several common allergenic foods, such as fish, milk, egg and nut [3]. Almost at the same time, Ortolani and collaborators [4] used the term OAS to describe a patient suffering from rhinoconjunctivitis, who showed oral symptoms after ingesting fresh fruits and vegetables. Subsequently, the presence of a new allergen of 13 kDa responsible for OAS was detected in some fruits. It was unrelated to pollinosis and was responsible for a cross-reactivity with fruits of the *Prunoideae* subfamily, such as peach, cherry, apricot and plum [5]. Therefore, it was clear that OAS could be induced by animal (egg, milk, seafood) [3, 6] and plant food allergens in the absence or presence of pollinosis.

**Fig. 1:**
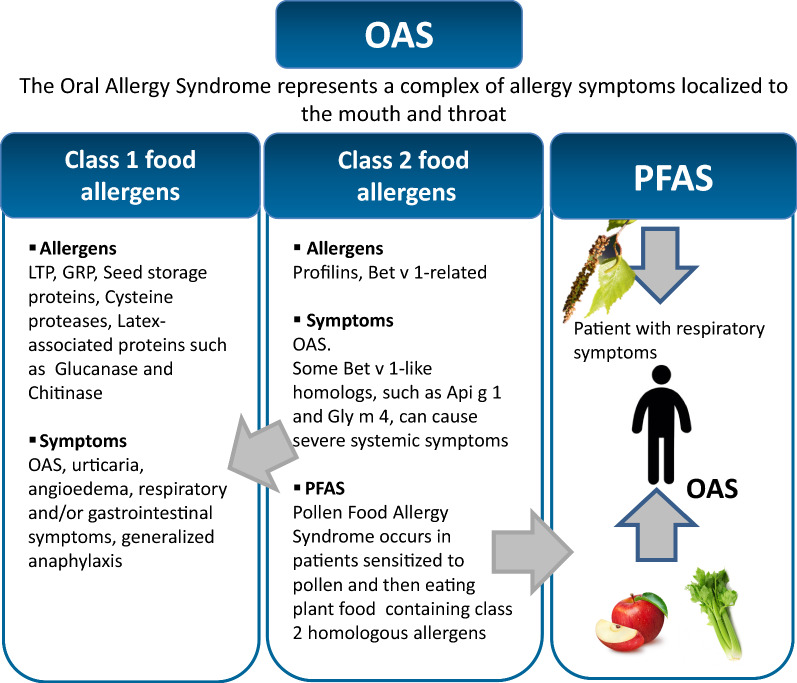
Overview of the relationships between OAS, PFAS, class 1 and class 2 food allergens

Conversely, the term “Pollen Food Allergy Syndrome” (PFAS) was proposed to define the oral symptoms following a primary sensitization to pollen allergens leading to an IgE co-recognition between plant aeroallergens and plant foods due to allergens belonging to the class 2 food allergy [7]. Therefore, the term PFAS should not be used as a substitute for OAS because the two terms define different processes (Fig. 1).

The advent of molecular allergology has rapidly increased the exploration of inhalant and food allergens in recent years, but the pathogenesis of allergic disorders is still obscure. An emerging hypothesis suggests that damage of the mucosal barrier could be the basis of OAS and PFAS. The airway epithelium represents a physical barrier defending subjects against inhaled harmful substances. Here the epidermal dendritic cells have a key function as inducers and silencers of allergic responses within the immunological network of mucosal surfaces [8, 9]. Furthermore, differences in oral bacteria (human salivary microbiome) could influence oral digestion and oral immune processes [10] as the presence of secretory IgA in the saliva could be a defense against harmful agents [11]. Likewise, such differences could induce IgA-mediated oral dysbiosis secondary to a dysregulation of intestinal microbiota.

In an attempt to unify symptoms and signs into a single entity, defined “syndrome”, several different associations of allergenic sources, often based on simple statistical calculations, have been progressively proposed. The molecular approach based on the identification of the allergenic proteins highlights how the sensitization to certain allergenic sources is highly dependent upon the patient’s peculiarities.

The aim of this article is to review remarkable clinical and molecular results related to OAS and PFAS induced by plant food allergens.

### Current diagnostic approaches

The diagnosis of OAS starts with an in-depth medical history. Skin prick test (SPT) and the serological testing of specific IgE (sIgE) are usually the first method of choice used for a preliminary screening of the source of allergic reactions. Nevertheless, SPT are usually performed using extracts that are problematic reagents providing not so reliable results. Definitely, double-blind, placebo-controlled food challenge (DBPCFC) remains the gold standard to diagnose food allergy in patients presenting OAS. However, this procedure is contraindicated in patients with past severe food responses because it can cause allergic reactions of unpredictable severity.

Basophil activation test (BAT) is a functional test useful for the diagnosis of pollen and food allergy and can be used to evaluate the possibility of more severe allergic reactions in patients with OAS [12, 13]. Actually, BAT is reported to be quite specific and able to estimate the threshold of allergic reactions, as well as to discriminate between sensitized and symptomatic patients. However, BAT is complex to be performed and therefore it is limited to selected investigations.

The search for specific IgE in the sera, using singleplex and multiplex systems, is clearly the easiest method to obtain a diagnosis and it is also completely safe for the patient. Serological tests can be performed using extracts or purified allergenic molecules as reagents. Unlike extracts, the allergen molecules represent standardized reagents able to provide indications on the patient sensitization to individual allergens. This knowledge is of great relevance in the management of patient suffering from OAS. In fact, the identification of allergenic molecules to which the patient is sensitized contributes to predict the possible allergenicity of, and cross-reactivity with, homologous molecules contained in other sources. This information can be used for the assessment of the risk of developing more severe reactions. For this reason the molecular-based approach is included in the context of precision medicine allowing a patient’s tailored personalized diagnosis.

## Strengths and pitfalls of diagnostic systems

### Tests in singleplex

Allergy tests in singleplex can be performed using protein extracts and purified allergens. Usually, on the basis of the clinical history, the allergist selects one or more allergenic sources and/or allergenic molecules to which the patient could be allergic. When the singleplex procedure is chosen, the number of allergens used for allergy diagnosis is restricted to “the most common allergenic sources”. For instance, Bet v 2 was recommended as a useful marker to detect sensitization to profilins in the sera of birch pollen allergic patients. Subsequent studies revealed species-specific IgE epitopes on profilins justifying the wide variation of the percentage of sensitizations to each profilin [14]. Therefore, following the singleplex approach, and much more the theory of the individual allergen as a marker for the entire protein family (including isoallergens, isoforms and homologous molecules) only fragments of information and incomplete profiles of the patient sensitizations can be achieved. The results obtained in such a way are sometimes difficult to be interpreted and can provide limited or inaccurate diagnostic conclusions that do not contribute to an understanding of processes such as cross-reactions, co-recognitions and PFAS (Fig. 1).

### Extracts for diagnosis

The commercial plant-derived food extracts for SPT and for the measurement of serum specific IgE are not biologically standardized. At least partially, this is due to the low abundance and/or lack of stability of the involved proteins. For example, the degradable conformational epitopes of Bet v 1-like proteins and profilins make them not always sufficiently represented in commercially available allergen extracts. Similarly, the prick to prick technique (PPT) is also not standardized and fresh food does not always contain all the allergenic proteins [15, 16]. This leads to a low specificity and sensitivity of the test. This is a drawback that cannot be overcome because it strongly depends on the high variability of the allergen panels contained in the starting materials. In fact, the variability of the allergen panels in the sources used for SPT or PPT strongly depends on the starting material. The low structural stability of some proteins is one of the involved factors. However, even more influential is the high variability of the expression levels observed for some proteins, depending on several factors, including the species, cultivar, climate conditions and chemical treatments. Some allergens are constitutively expressed proteins (CEP) [17] and are present in a quite constant amount in the natural source. A well known example is Pru p 3 that is found in high amounts in the peel of every peach cultivar, and in every fruit batch independently of the cultivation method, the climatic conditions and the applied chemical treatments [18]. In contrast, other allergens are factor-induced expressed proteins (FEP), such as ENEA that is found in very variable amounts in the allergenic source (peach), ranging from the complete absence to the presence in a high concentration [17]. FEP allergens may affect the prevalence of sensitized subjects and could contribute to generate an intermittent clinical reactivity to the allergenic source. In addition, FEP allergens represent an important cause of the variability of extract composition [19], including the extracts used for diagnosis, contributing to impair the standardization of these reagents. For this reason the use of FEP allergens as purified molecules in testing systems appears of great importance considering that the standardization of extracts, but also of the allergenic sources used to produce the extracts available for allergy diagnosis, is not possible.

### Tests in multiplex

Allergists have to deal with molecular mechanisms of allergy and need to characterize the endotype of every single patient renouncing the idea that a patient, on the basis of his/her history, should be tested only towards a few molecule markers coming from “the most common allergenic sources” [20]. Only tests in multiplex can provide a comprehensive profile of the patient sensitizations. In addition, the use of molecular allergy diagnosis can map the exact allergen sensitizations of patients. However, as many allergenic proteins have not yet been identified, or are not yet available for diagnosis, the use of extracts can at least partially be integrated with the diagnostic results. On the basis of these concepts, some years ago the FABER test was released and represented the new generation allergy test, a great leap in quality compared to the ISAC test [21] which was widespread at that time and which uses only allergenic molecules (Thermo Fisher Scientific, Phadia, Sweden). FABER is the first multiplex allergy diagnostic test that combines allergenic molecules and extracts to achieve the best possible diagnosis. This system also has the advantage of being implemented in the framework of a research and development project that provides it with ongoing improvements as research acquires new knowledge in the specific field. This feature allows FABER to have exclusive allergens, not included in other allergy tests, because they result from constant translational allergological research based on the three fundamental pillars “bench to bedside, and to community”. Since FABER has proved to be a great step forward in the field of allergy diagnosis, it has been used as a reference for the production of another test system [22] based on the use of a combination of molecules and extracts, namely ALEX (Macroarray Diagnostics, Vienna). Nevertheless, FABER and ALEX remain different systems for several reasons, including the selection of allergens used for diagnosis, the choice of the mode of the test execution and the type of support to the clinicians [20]. In addition, as literature reports highlight [17, 23–26], FABER is suitable for experimental investigations. Its centralized execution allows the collection of data about large populations of different geographical areas. The collected data can be compared because they are not affected by operator and experimental condition variations. In the future, the collection of data coming from large random populations and selected sub-populations will provide precious epidemiological information also useful to better understand cross-reactions, co-recognitions and syndromes. Furthermore, the technical execution of the FABER test allows the implementation of inhibition tests thus contributing to an increase in the knowledge in the field of molecular allergology by providing data useful to identify and characterize new allergens. For instance, the first data on the cross-reaction between GRP allergens were obtained with the FABER test, that nowadays is the only allergy diagnostic test including Pru p 7 and Pun g 7 in the standard biochips and also Cup s 7 for experimental purposes in some additional tests [24]. Additionally, this test has provided data on the sensitization prevalence and cross-reactions between chitinases belonging to different classes, namely chitinase I, III and IV from latex, pomegranate and kiwifruit, respectively [25].

Beyond the test system used, what is very important is that the IgE test results must always be interpreted in the context of the patient’s clinical history and all his/her allergic sensitizations should be taken into account. It is very dangerous to reduce the observed allergological phenomena to the very small group of diagnoses known so far.

## Structural similarities in plant allergenic proteins

Plant species share many proteins, including allergenic ones. A large amount of data coming from decades of studies in the field of molecular biology and genome sequencing shows an increasing number of proteins potentially expressed in all plant species. In fact, many genes coding for homologous proteins have been detected in the genomes progressively investigated. Some of these proteins are always found in all the analyzed plant organisms, whereas others are not routinely detected, but sometimes are observed. Therefore, even proteins never detected in specific plant organisms could be present and expressed in some conditions. Generally, proteins involved in the basic mechanisms of the organism physiology, common to all plant species, are those showing homologs in all plant species.

**Fig. 2:**
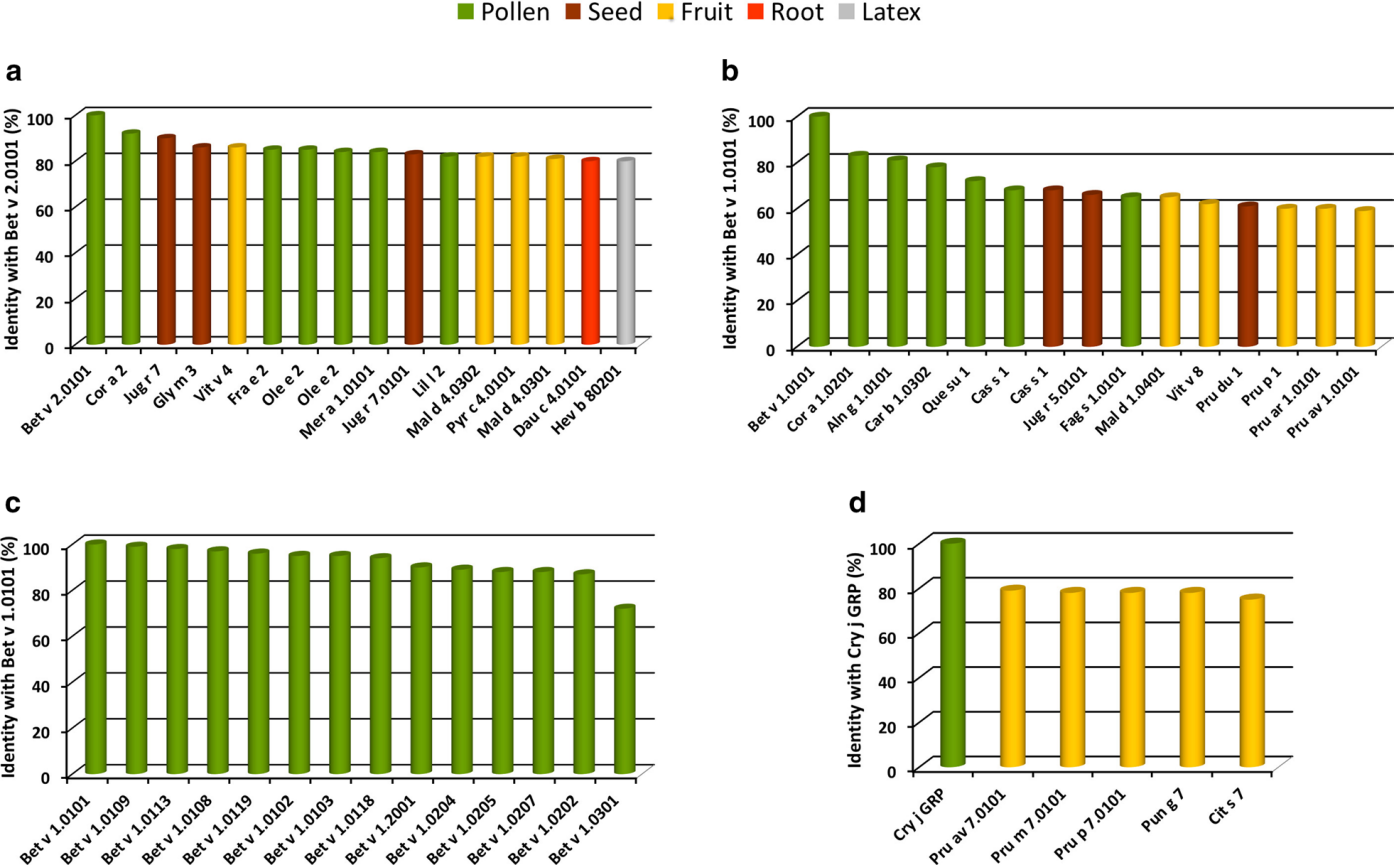
Comparison of amino acid sequence identity percentage between different allergens. Bar colors highlight the plant source of allergens, namely green, brown, yellow, red and light grey are for pollen, seed, fruit, root and latex, respectively. The similarity searches were performed with the Allergome Aligner tool, using the Blast algorithm on the Allergome platform (https://www.allergome.org). The amino acid sequence of Bet v 1.0101 (**b** and **c**), Bet v 2.0101 (**a**) and Cry j GRP (**d**) were used as query sequences. The allergens shown in **a** and **b** were selected as representative of the large number of homologs found in the database. When many homologs were found, that giving the highest identity with the query sequence was selected and shown in the figure. **c** represents a representative selection of Bet v 1 isoallergens/isoforms found in the Allergome database. The sequence identity values of GRP (**d**) were obtained with the algorithm CLUSTALO on the Uniprot website (https://www.uniprot.org) and they are referred to mature proteins lacking the signal peptide. Cup s 7 was not included because its complete sequence was not available. Additional details on the allergens shown in the Figure are reported in Additional file 1: Tables S1, S2, S3 and S4

Profilin is an example of an allergenic protein found in all plants and also in all other eukaryotic organisms and viruses. Profilins play a key role in cell physiology and show a high conservation of their structural features [27] highlighted by the high amino acid sequence identity between homologous molecules (Fig. 2a and Additional file 1: Table S1). In fact, Fig. 2a shows that sequence identity values in the range from 80 to 100% were observed when the primary structure of birch pollen profilin, Bet v 2.0101, was compared with those of homologs found in different plant tissues and species belonging to various taxonomic orders (Additional file 1: Table S1). This very high sequence identity is far greater than that (35%) suggested by the FAO/WHO/EFSA/Codex as a value predicting a possible allergy risk [28]. Therefore, this strongly suggests that patients sensitized for instance to the pollen profilin Bet v 2 have a high probability of having IgE co-recognizing the homologs contained in sources such as hazelnut and olive tree pollens, walnut and soybean seeds, grape, apple and pear fruits, carrot root and latex (Fig. 2a). Quite a high sequence identity is also observed when other allergens such as those belonging to the Bet v 1-related (Fig. 2b and Table Additional file 1: S2) and gibberellin regulated protein (GRP) families are compared (Fig. 2d and Additional file 1: Table S4).

The high sequence identity suggests that the IgE epitope panel should be at least partially shared between the homologous allergens. Some IgE epitopes could be common to different homologs. Conversely, others could be peculiar to individual species and could provide a monosensitization to that source without any cross-reactions with the homologs contained in other sources, as reported for instance for lipid transfer proteins (LTP) [29, 30]. This is in line also with reports describing the so-called PFAS where the prevalence of patients reacting with both pollen and food homologous allergens (such as profilin and Bet v 1-related), is always less than 100%. This means that to predict the cross-reaction between homologous molecules, and therefore the sensitization to the sources of the homologs, it is not sufficient to know the allergenic molecule. In the future, the next step should be the detection and discrimination between different specific epitopes recognized by the IgE and then the definition of the individual panel of epitopes that an allergen shares with each homolog.

Indeed, a prediction of sensitization to different sources containing homologous allergens is complicated further by the presence of isoallergens and isoforms expressed in the same source, and within the individual organism, which can differ in their allergenic properties [31]. In order to make the classification of homologous allergenic molecules clear, recently, the WHO/IUIS Allergen Nomenclature Sub-Committee has ruled that sequences within about 67% identity to the original allergen are designated as isoallergens and sequences differing by < 90% identity are isoforms or variants. Isoallergens are designated by the addition to the allergen name of two digits after the decimal point in the number and isoforms or variants by the addition of two more digits (e.g., Amb a 1.0101) [32].

It is known that allergens such as profilins, Bet v 1-like and LTP belong to multigene families coding for many isoallergens/isoforms. Figure 2c and Additional file 1: Table S3 show just one example of a few Bet v 1 isoallergens/isoforms ordered on the base of their decreasing sequence identity with Bet v 1.0101. The molecules shown in the figure are representative of the 38 present in the Allergome database [33], after a similarity search using the Allergome aligner tool and Bet v 1.0101 as the query sequence. Therefore, the presence of isoallergens/isoforms enlarges the IgE epitope collection associated with an individual allergen. For instance, Jimenez-Lopez and collaborators [34] highlighted how a conspicuous variability in linear and conformational epitopes between profilins belonging to the same olive cultivar, and among different cultivars, was found as a direct implication of the sequence polymorphism. Therefore, these aspects should be kept in mind when topics such as allergy diagnosis and cross-reactivity between homologous allergens are analyzed. We cannot forget that different factors, such as the species, cultivar, tissue, geographical location, climate environment and chemical treatment, can affect the IgE epitope panels of an organism by modulating the number and type of allergens and isoforms expressed in the allergy source [31, 35, 36]. It is worth noting that a few studies report also IgE cross-reactivity between unrelated allergens [37]. However, in these studies the structures comparison showed that the cross-reactivity between nonhomologous allergens was based on highly similar and surface-exposed stretches of protein sequences. For instance, this is the case of IgE binding to the 2S albumin Ara h 2 that was completely inhibited by the cupins Ara h 1 and Ara h 3 [38]. Therefore, similar to homologous allergens, the cross-reactivity between nonhomologous ones is based on the presence of common structural features.

In addition, looking for possible interpretations of unclear results, we should always remember that the allergy diagnosis is very often performed using only one of the possible isoallergens and/or isoforms that an allergen, such as profilin, Bet v 1-like and LTP, can express in a species. This implies that the IgE epitope panel used for diagnosis is limited because it is associated with an individual molecule, meaning that unshared epitopes, specific of other isoforms, are missed. This can generate false negative results. It can also happen that a patient testing positive with an allergen isoform, has indeed IgE recognizing epitopes that are not shared with all the other isoforms. This patient will not display allergic symptoms if he/she is exposed to pollen or food sources that express isoforms with an epitope panel for which he/she does not have specific IgE.

## Class 2 food allergy

**Fig. 3:**
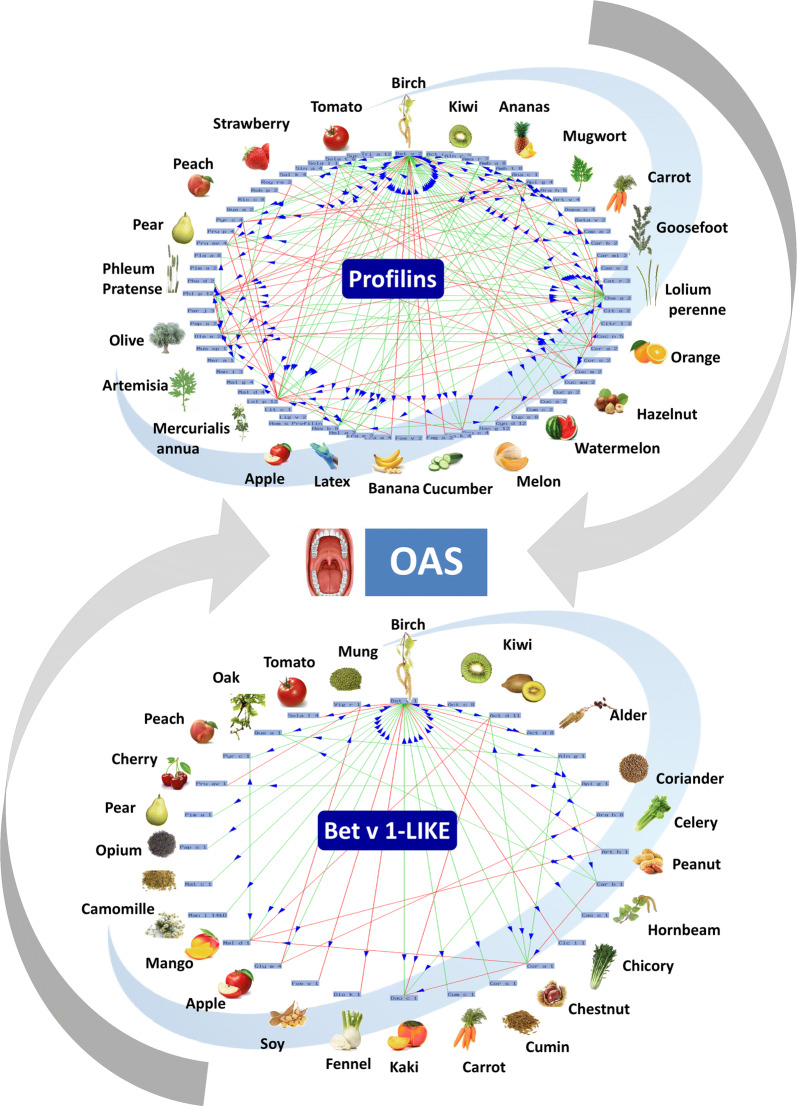
Schematic representation of cross-reactivity occurring between profilins (upper O-ring) and between Bet v 1-related allergenic proteins (lower O-ring) contained in pollens and plant foods. Red and green lines connecting allergy sources indicate single and double cross-reaction relationships, respectively. Arrowheads indicate the direction of data collection. The two pictures were generated on January 2020 by the Allergome software which uses information contained in the own database [53] (https://www.allergome.org)

Class 2 food allergens are generally heat-labile proteins, susceptible to digestion, and structurally similar to pollen homologs [39] (Figs. 1, 3). Profilins and Bet v 1-like proteins are classified as class 2 food allergens, responsible for OAS but not for systemic reactions, since they are generally readily denatured or degraded by digestive enzymes and/or heat [40]. However, some seed Bet v 1-like proteins, such as Gly m 4 and Ara h 8, depending on the exposure conditions, can show remarkable stability [41, 42]. Furthermore, the significant damage to the epithelial barrier of the oral mucosa was recently described in profilin allergic patients. It could allow the crossing of the allergen into the oral mucosa eliciting local inflammation and facilitating the increased sensitivity of effector cells [8, 9].

Patients suffering from pollinosis may display PFAS when ingesting some plant-derived foods (Fig. 1). This syndrome is caused by IgE cross-reactive molecules, profilins and Bet v 1-related proteins, shared by inhalant and food allergen sources. PFAS is characteristically manifested as OAS. The prevalence of PFAS affected patients varies with the type of allergen eliciting symptoms and the country where the reaction occurs. In fact, the culprit food might be also correlated to geographical eating habits. Moreover, the local pattern of foods causing PFAS may change when new foods start to be consumed outside their native area. So far, no accurate statistics on the frequency of PFAS are available. Additionally, the degree of clinical reactivity may show seasonal variations [43]. In line with these observations, PFAS has been defined as a “complex syndrome posing a diagnostic and therapeutic challenge” [44]. In fact, some aspects are still unclear and the positive IgE testing to profilins and/or to Bet v 1-like proteins does not always correspond to clinical reactivity, that is some patients sensitized to pollen allergens react to plant foods after exposure while others do not [45]. Probably, more in-depth investigations at the molecular level could shed some light on this complex process. For instance, although literature reports highlight the possible different immunological behavior of the isoallergens and isoforms of a specific allergen [29, 32, 34, 46, 47], this aspect is often underestimated, or even forgotten, when diagnostic and clinical results have to be interpreted. In this context, it could be of interest to understand, for each allergen, to what extent the panel of IgE epitopes contained in the allergen molecule used for diagnosis is shared with isoallergens and/or isoforms found in different cultivars of pollens and plant foods. Moreover, useful indications could come from a survey of the plant cultivars adopted in combination with different climate conditions and geographical locations of the cultivations producing pollens and plant foods. In fact, we cannot exclude the possibility that a patient sensitized to allergens coded by multigene families, such as profilin [48] and Bet v 1-related [47], can have personalized sets of IgE which recognize epitopes borne by a limited number of isoallergens and/or isoforms. In this case, depending on the allergen isoform used for the diagnosis and on the features (including cultivar, climate and chemical treatment) of the allergen source (pollen and food) to which the patient is exposed, we could obtain different combinations of results. For instance, we might observe that (i) the patient is tested IgE positive and shows symptoms after exposure to pollens and foods, (ii) the patient is tested IgE positive and reacts to only one of the allergen sources (pollen or food), (iii) the patient is tested IgE negative but reacts to both pollen and food sources or (iv) the patient is tested IgE negative and reacts either to pollen or to food.

Despite only two main protein clusters of panallergens being involved in the classic framework of PFAS, several clinical syndromes have been described over time. For instance, those associating birch and apple, birch, apple and carrot, birch and hazelnut, birch and apiaceae, ragweed, mugwort and birch, celery, mugwort and spice, mugwort and peach, mugwort and chamomile, ragweed, melon and banana and goosefoot and melon. It seems clear that memorizing the ever-increasing list of names rather than understanding the underlying food derived allergenic proteins could lead to unnecessary confusion. A syndrome is a set of symptoms that can be caused by allergens that are not homologous contained in the different allergen sources. Therefore a long list of syndrome names may hinder rather than help the allergist because he/she may stop searching for the allergens responsible for the symptoms.

### Profilins

Profilins are actin-binding proteins, present in the cytoplasm of all eukaryotic cells, found in both animal and plant sources. Although attempts to estimate the profilin concentration in allergenic sources have been reported [49], the analysis of its amount is not easy because this protein is present in the cells as a free molecule and a molecule bound to actin and other ligands [50]. Up to now more than 120 allergenic profilins have been described from different taxonomical species (https://www.allergome.org).

Profilins are heat-labile proteins and they are easily degraded by the gastrointestinal proteases. Although the primary structure is highly conserved, profilins from different sources show some individual structural features, as also highlighted by the different melting temperatures reported for some of them deriving from grapevine, bell pepper, watermelon, hazel, muskmelon, soybean, apple, bean, cherry, almond, peach, tomato and potato [51].

The primary structure of profilins comprises 125–153 amino acids and their isoelectric point ranges from 4.3 to 9.2. In line with the crucial role of profilins in the plant physiology, generally a high similarity of the primary structure is observed when the proteins of different plant species are compared (Fig. 2a). Even more highly conserved than the amino acid sequence is the overall 3D-structure of these proteins. In fact, their folding is found conserved even in profilins with a low sequence similarity and in phylogenetically distantly related, or unrelated, organisms [51, 52]. Therefore, the common co-recognition between different profilins [53] can be related to a shared panel of IgE epitopes due to the conservation of sequence fragments and the overall folding [34]. Nevertheless, the presence of common epitopes does not prevent the possibility that some additional ones can be generated by a few amino acid substitutions and associated to individual isoforms, or to a group of them, but not shared with all the profilins.

Due to their ubiquitous presence, profilins are panallergens, eliciting IgE responses in about 10–20% of pollen-allergic patients. In fact, depending on the geographical location of the analyzed populations, the prevalence of sensitized patients is reported to be different, ranging from approximately 5% in Swedish birch pollen–allergic patients [54] to 20–30% in populations living in Austria, France, Portugal and Italy [55–57] and 60% in Spain [58].

The plant foods more often associated with profilin hypersensitivity are muskmelon, watermelon, citrus fruits, banana, pineapple, tomato, persimmon and zucchini. Several studies have also reported profilins as responsible for the fruit–fruit, fruit–pollen and latex–food syndrome [59, 60]. Muskmelon has been suggested as a marker of food profilin recognition [61], although it contains genuine allergens, such as Cuc m 1 [62]. Therefore, as a general rule, it should never be forgotten that an allergenic source can contain additional and also genuine allergenic proteins, sometimes not yet identified, that can cause allergic reactions. In such cases, it can be difficult to discriminate between the symptoms caused by one or another culprit.

Some studies described patients suffering from generalized reactions to profilins [63, 64]. For instance, Fah and collaborators described a patient suffering from an inhalant allergy to mugwort pollen who had OAS and anaphylaxis after eating lychee fruit and sunflower seeds, assigning symptoms to profilins [63]. Reindl and collaborators described a systemic reaction induced by raw and cooked zucchini in four patients; three of whom also having OAS. The systemic reaction was attributed to profilins in three of these patients while in the fourth the culprit protein was not identified [64]. The possibility cannot be excluded that data obtained many years ago, when the number and type of known allergens were very limited, could have led to imprecise or incorrect interpretations. Probably, the same data would be interpreted in a different way nowadays, and even more precisely in the future, as knowledge progressively increases. The use of allergy tests providing comprehensive and personalized profiles of sensitizations, combined with a careful interpretation of the diagnostic results, is always necessary in order to confirm the allergy without stopping at the first appearance as “all that glitters is not gold”.

More recent studies have suggested that sensitization to profilins would protect against stable allergens, such as LTP and seed storage proteins causing systemic reactions [65–67], although the reason for this is not known. However, more longitudinal studies are required to understand if the OAS induced by profilin comes before LTP sensitization. In this case, the OAS induced by profilin might protect patients by inducing them to avoid the ingestion of foods containing the more dangerous LTP, thus preventing the sensitization to this class 1 allergen.

### Bet v 1-related allergens

Bet v 1 and its homologs are members of the family of the pathogenesis-related proteins 10 (PR-10). These proteins protect plants against attack from different pathogens, which can induce an increased expression of the molecule. Bet v 1-like proteins of different plant species share a molecular weight of about 17 kDa and their primary structure is quite conserved [68]. The 3D-structure is even more highly conserved than the amino acid sequence. The importance of the protein 3D-structure, and of its conformational epitopes, in the IgE co-recognition of Bet v 1-like proteins, is highlighted by the Act d 11 cross-reactivity with the Bet v 1-related allergens. The kiwi allergen Act d 11 belongs to the Ripening Related Proteins/Major Latex Proteins (RRP/MLP) family [69]. It shares the same folding with Bet v 1-like proteins [70] and shows an IgE cross-reactivity with them, including Bet v 1, despite their very low sequence identity. The mung bean Vig r 6, belonging to the Cytokinin-Specific Binding Proteins (CSBP) subfamily [71] is an additional example of a protein not belonging to the PR-10 family cross-reacting with the Bet v 1-related allergens [68]. Tree pollens containing Bet v 1-like proteins, such as birch (Bet v 1), alder (Aln g 1), hazel (Cor a 1), hornbeam (Car b 1), chestnut (Cas s 1), beech (Fag s 1) and oak (Que a 1), are responsible for allergic respiratory symptoms in humans. The prevalence of sensitization to these allergens is higher in north and mid-Europe and lower in the south. After a primary sensitization, some birch pollen-allergic patients develop OAS at once or within minutes after the ingestion of fruit, nuts and vegetables containing Bet v 1-like proteins.

As they belong to the class 2 allergen group, it is most likely that the Bet v 1-like proteins induce symptoms only after pollen sensitization (Fig. 3), at least those labile to pH changes, heat treatment and digestion [72]. However, some seed Bet v 1-like proteins can show remarkable stability. For instance, Ara h 8 and Gly m 4 are Bet v 1-homologous and they are major allergens in patients with combined birch pollen and peanut or soy allergy. They both are potential triggers of generalized severe reactions. Processing and ligand interaction sometimes can increase their stability [73]. For instance, roasting and lipid binding provide allergenic and proteolytic stability to Ara h 8 [41] and the type and degree of processing can influence the allergenicity of Gly m 4. In particular, the highest amount of Gly m 4 was observed in dietary powders and in soy drinks. Therefore not all soy-based foods are able to cause the same allergic reactions [42].

Moreover, the amount of some Bet v 1-like proteins in the allergenic source varies with the ripening time [74] and cultivar type [15]. In most cases, they cause mild oral symptoms. However, all patients report the absence of reactions when they eat cooked, canned, and preserved foods containing Bet v 1-related allergens. In addition, 37% of patients suffering from eosinophilic esophagitis described OAS induced by Bet v 1-like-containing sources [75].

Sensitization to food Bet v 1-like proteins without pollen sensitization has also been reported [76, 77]. This lack of sensitization to pollen could be only apparent due to the expression of subtypes of IgE antibodies specific for epitopes borne on different Bet v 1-like isoallergens/isoforms. In fact, while the Bet v 1-like proteins of the *Fagaceae* pollen are generally cross-reactive, only 25% of the IgE epitopes of the *Betuloideae* and of the *Coryloideae* pollen allergens are exclusive for the respective subfamily [78]. Therefore, the measurement of Bet v 1-specific IgE alone may not be sufficient to reach a diagnosis, especially in a birch-free area [79]. In addition, it has been noted that not all pollen sensitized patients with Bet v 1-related food allergy suffer from spring rhinitis [80].

The symptoms induced by food Bet v 1-like proteins are not usually severe, although some anaphylactic reactions to soy [81], celery (Api g 1), carrot (Dau c 1) [82, 83], jackfruit and sharon fruit [84, 85] or to different mixed vegetables have been described [86]. Actually, in most cases it was not possible to discriminate whether the reaction was caused by Bet v 1-like proteins or by other allergens. Some of these reports are limited since they base their results exclusively on the patients’ history of known birch pollen allergy and on testing for a limited number of allergens. Nevertheless, in some cases the sensitization to a specific allergen, such as Gly m 4 from soybean, was linked to the development of severe and generalized allergic reactions upon soy consumption [81]. More immunological studies are needed to better understand some results obtained in the absence of appropriate purified allergens.

Apple, peach and other fruits, as well as tree nuts and peanuts, are the most common foods eliciting allergic reactions in Bet v 1 like sensitized subjects with differences in prevalence related to different geographical areas [87, 88] and to the patient specific profile of IgE recognition [78]. Similarly to the described sensitization to profilins, the sensitization to Pru p 1 (Bet v 1-like of peach fruit) has been suggested to be associated with a lower risk of severe reactions to peach LTP [65].

The discrimination between OAS positive and OAS negative patients sensitized to Bet v 1-like allergens could be achieved by an analysis of the patient’s phenotype. In fact, it has been suggested that patients with high levels of Bet v 1-like specific IgE display OAS more frequently than other allergic subjects. On the other hand, OAS negative patients display a broader sensitization pattern towards other inhalant allergens [89].

## Class 1 food allergy

**Fig. 4:**
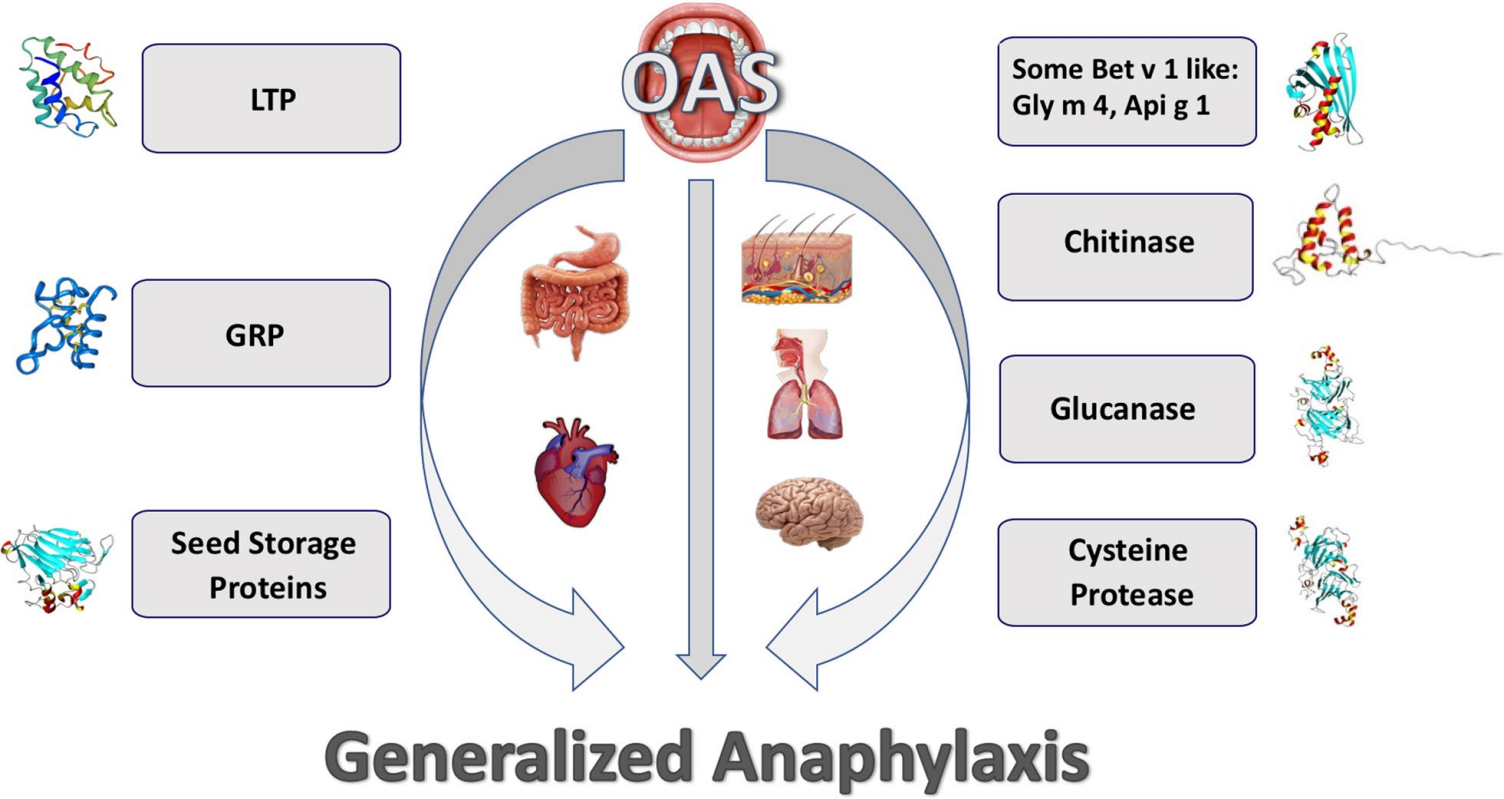
Schematic representation of the class 1 plant food allergens that may cause OAS and/or trigger allergic reactions in other organs and apparatus. These allergens may even induce severe anaphylaxis

Class 1 food allergens (Figs. 1, 4) induce allergic sensitization via the gastrointestinal tract and are responsible for systemic reactions as they are resistant to gastrointestinal digestion and heat [39].

### Lipid transfer proteins (LTP)

LTP are a family of ubiquitously expressed small plant proteins belonging to the prolamin superfamily. They are members of the family of PR-14 and are involved in plant defense from biotic and abiotic stress. LTP can bind a broad spectrum of lipids, thus functioning as intra- and extracellular carriers for hydrophobic molecule transportation [90]. It has been suggested that the conjugation of lipids to LTP may induce structural modifications that may affect the IgE-binding capacity of the protein [91]. Plant LTP can be divided into two subfamilies according to their molecular weight, 9 kDa LTP (9 k-LTP) and 7 kDa LTP (7 k-LTP) [30]. Their highest expression levels have been found in peripheral cells associated with the cell wall and cuticle of epidermal tissues while the pulp contains levels much lower than the peel. LTP are resistant to pepsin digestion and heat due to their compact cysteine-stabilized folding. In a neutral environment they show a much lower resistance to heat treatment than at a more acidic pH [92, 93].

Pru p 3, the peach LTP, was the first one to be characterized in edible plant food [94] with a high prevalence of sensitization in Italian and Spanish populations [14, 95]. Later, a sensitization with different prevalence values was described for several populations living in different geographical areas [96–98]. Pru p 3, being the most recognized allergen of this family, was proposed by some authors as “the marker” of LTP sensitization. Nevertheless, this view is now outdated because a more in-depth analysis of molecular and clinical data has shown that sensitization to LTP can occur in the presence or absence of sensitization to Pru p 3, as in the case of Act d 10, the kiwi LTP, or Pun g 1, the pomegranate LTP [29, 99].

OAS is probably the most frequent clinical symptom of LTP allergy. Often, but not always, OAS is associated with gastrointestinal symptoms and/or generalized reactions, occurring with no correlation between specific IgE levels and clinical severity [100, 101]. The severity of allergic reactions could depend on the specific LTP sensitizations of the patient and on the eaten source [102]. However, the sensitization to LTP does not necessarily imply allergy. In fact, some patients tolerate foods to which they are sensitized, while others have been reported to react only in the presence of co-factors, such as NSAIDs, alcohol intake and exercise [103, 104]. Some patients suffer from severe allergic reactions despite having low specific IgE levels. To date, fatal reactions induced by LTP allergy have not been reported.

Cross-reactivity among allergenic LTP contained in foods has been described, even between members of botanically unrelated species. The WHO/IUIS Allergen Nomenclature Sub-committee lists 23 different LTP contained in plant foods. Twenty-six additional LTP are listed in the Allergome database (https://www.allergome.org) as responsible for allergic reactions to foods including rice, barley, fennel, parsley and carrot. LTP are panallergens contained in so many and so different sources as to make the differentiation between co-recognition and co-sensitization unfeasible. Cross-reactivity between plant food LTP and the pollen homologs has been reported to be low in the case of Artemisia (Art v 3) and Platanus (Pla a 3), and even absent in the case of Parietaria (Par j 2) and Olea (Ole e 7) [105]. This is probably related to the low sequence identity between these proteins and their different lengths. Art v 3 has been proposed as a sensitizer allergen in mugwort-allergic patients with IgE recognizing Pru p 3, due to a high exposure to mugwort pollen present in some countries [96]. Other authors suggest that the sensitization to Art v 3 could depend on shared epitopes with Pru p 3 in the absence of a real allergy to Artemisia pollen [106]. The variable geographical prevalence of Artemisia can in part account for the differences in the study results. Additionally, inhibition studies, using ImmunoCAP and ELISA, have shown how Art v 3 significantly inhibits the binding of IgE to Pru p 3. Conversely, Pru p 3 did not inhibit IgE binding to Art v 3 [96, 107]. In addition, no IgE cross-reactivity between Pru p 3 and Art v 3 was observed in mediator release assays [108].

Similarly, no general conclusion on the primary sensitizer between the pollen Pla a 3 and the fruit Pru p 3 can be drawn as they have been reported to show different biological activities in histamine release assays depending on the sera of the individual patients tested. Some evidence for an IgE cross-reactivity between Pla a 3 and Pru p 3 has been detected. In fact, Pru p 3 was observed to inhibit IgE to Pla a 3 but not vice versa [109], thus supporting the idea that the peach LTP is the main primary sensitizer and the IgE recognition of the plane tree homolog is due to cross-reactivity. IgE specific to Pla a 3 could identify the subgroup of plane tree pollen allergic patients predisposed to allergy to food LTP [110]. Recently, new insights into the possibility that the olive LTP, Ole e 7, could play a role as a primary sensitizer in regions with a high olive pollen exposure has been confirmed [111].

It has been hypothesized that the co-recognition between inhaled and food LTP could have a spreading role. This theory should be further developed and explored considering that the wider is the patient’s LTP sensitization, the worse appears the prognosis [112]. In summary, some studies suggest that the co-sensitization to LTP and profilin and/or to Bet v 1-like proteins could have a protective role to prevent systemic reactions [65, 66], whereas others conducted in different populations do not confirm this hypothesis [113]. Other authors even report opposite results showing that in Chinese LTP allergic patients the sensitization to profilins rather remarkably increases the risk of systemic symptoms [114]. Therefore, it appears evident that larger longitudinal studies on large populations of different ethnicities are required to reach definitive conclusions.

### Gibberellin-regulated proteins (GRP)

Peamaclein (Pru p 7) is the first allergenic GRP identified in 2013 as a fruit allergen causing OAS and anaphylaxis [115]. It is a tightly structured α-helical protein of about 7 kDa, with an electrophoretic mobility very similar to that of the 9 k-LTP Pru p 3. Pru p 7 is present in significant concentrations in both peach peel and pulp. It is resistant to intestinal digestion and has a significant thermal resistance beginning to unfold over 100 °C. In addition, it has been observed that heat-denaturation modifies the immunological properties of Pru p 7 [116]. The amino acid sequence of GRP is very well conserved (Fig. 2d) through several botanical species [115]. After Pru p 7, Pru m 7 from Japanese apricot, Pun g 7 from pomegranate and Cit s 7 from orange have been identified as allergens and described as a cause of allergic symptoms, including OAS and anaphylaxis [23, 117]. Cypmaclein, a Pru p 7 homolog from cypress pollen, has recently been identified as the first inhalant allergen of the GRP family [24]. GRP belong to the snakin/GASA family and their expression is up regulated by gibberellins (GA), which are a class of natural phytohormones. GA and GRP play a crucial role in plant growth and developmental processes. Some gibberellins, for instance GA3, represent some of the best-selling and most important plant growth regulators. They can be obtained in liquid, soluble powder, wet table powder, tablet or water-dispersible granular forms. The external application of synthetic GA, generally by spraying, is a widespread agricultural treatment used to increase and improve crop production. For example, gibberellins are used to delay citrus maturity, to fatten seedless grapes, to increase cherries in size, weight, and firmness and to increase the quantity of malt for brewing or sucrose in sugar cane. However, the product’s cost could limit its constant application [118]. This treatment might influence the amount of GRP produced in plant-derived foods, but more studies are required to verify if such treatment influences the allergenicity of the crops.

PFAS has been described in cypress pollen allergic patients living in Southern France [119, 120]. They reported oral symptoms, pruritus and angioedema during ingestion of peach and/or citrus. The presence in cypress pollen of a new basic protein (BP14), with an apparent molecular mass of 14 kDa, was reported to be able to discriminate between patients allergic only to cypress pollen and those with an associated sensitization to Pru p 3 [121]. Years later, thanks to the discovery of the peach GRP, Pru p 7, by Tuppo and collaborators [115], the French researchers [119] observed the existence of an IgE cross-reactivity between BP14 and the recombinant Pru p 7. Senechal et al. also identified a fragment of BP14 showing a structural similarity with a stretch of the Pru p 7 sequence [119]. In this way, they described the clinical relevance of BP14 in peach PFAS [119, 120] and suggested that the sensitization to Pru p 7, and to the allergenic homologs, could be subsequent to a sensitization to the cypress pollen GRP, due to the large amount of Mediterranean *Cupressus sempervirens* pollen in Southern France [122]. It is important to stress that BP14 sensitization is independent of the sensitization to group 1 and/or 2 of *Cupressus* allergens [123].

In 2019, Tuppo and collaborators isolated the GRP protein contained in the *C. sempervirens* pollen, naming it cypmaclein [24]. Its structural and immunological features were compared with those of the homologous GRP from peach, Pru p 7, and from pomegranate, Pun g 7 [23]. Cypmaclein displays the same molecular mass as Pru p 7 and other GRP, that is about 7 kDa. In IgE inhibition experiments with the FABER system, using sera of patients sensitized to Pru p 7, cypmaclein showed a cross-reaction with Pru p 7 and Pun g 7. Subsequently, cypmaclein has been registered by the WHO/IUIS Allergen Nomenclature Sub-Committee as Cup s 7.

**Fig. 5:**
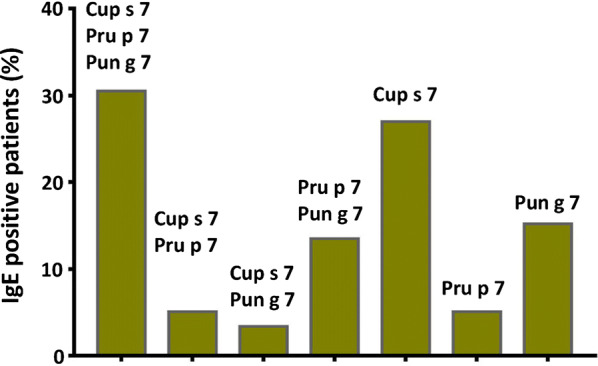
Sensitizations detected in 39 patients IgE-positive to at least one of the three analyzed GRP. The columns indicate, in order, patients sensitized to all the three GRP, those bi-sensitized to Cup s 7 and Pru p 3, to Cup s 7 and Pun g 7, to Pru p 7 and Pun g 7 and those monosensitized to Cup s 7, to Pru p 7 and to Pun g 7

The combination of the literature reports with the results of the study by Tuppo and collaborators strongly suggests that the cypress protein that was named BP14, and proposed to cross-react with GRP, is cypmaclein. In order to verify these results in real life the sera of 74 patients were tested on a set of experimental FABER biochips [124]. Based on the obtained results the patients’ sera were divided into two groups, Group A with 39 patients sensitized to Cup s 7 and Group B with 35 patients negative to Cup s 7. In Group A, 59% of the patients sensitized to Cup s 7, cypmaclein, proved to be IgE positive to at least one of the two fruit GRP. In Group B, a similar percentage (57%) of the patients negative to the cypress GRP proved to be positive to at least one of the two fruit GRP. Among the 74 patients, 59 were positive to at least one of the three analyzed GRP, Cup s 7, Pru p 7 and Pun g 7. It is worth to note that, out of these 59 patients, about 30% of them were sensitized to all the three GRP, whereas those monosensitized to Cup s 7, Pru p 7 and Pun g 7 were 27%, 5% and 15%, respectively (Fig. 5). Therefore, in the analyzed patients, the sensitization to the fruit GRP appeared not to be dependent upon sensitization to the cypress GRP, thus indicating that some IgE epitopes are not shared between all these molecules, despite their high sequence identity (Fig. 2d). Examining unselected samples, an IgE reactivity to three, or even one, GRP proteins, was found. As a rule of thumb, before concluding that an allergenic source, such as cypress pollen, might induce sensitization to another source, such as peach or pomegranate fruit, all the involved allergenic proteins should be carefully searched and analyzed [124], according to the concept “one does not fit all”, already described for other allergens [20, 29].

On a speculative basis, co-recognition of different GRP from different sources (plant food and pollen) might enhance the immune response in a synergistic manner and potentially lead to epitope spreading. Since not all patients sensitized to the Mediterranean *C. sempervirens* pollen are sensitized to the food GRP, the unsolved question is whether allergy to inhaled Cup s 7 is able to cause GRP co-recognition in food or vice versa. Further longitudinal studies on GRP IgE reactivity are required to understand if a sensitization to these allergens starts from cypress pollen or, rather, it is secondary to sensitization to plant food containing GRP. It should be equally important to understand whether or not patients monosensitized to Cup s 7 suffer from respiratory symptoms.

### Latex-associated plant food allergy

Up to 30–50% of latex allergic patients show signs of an associated hypersensitivity to various fresh fruits (avocado, banana, chestnut, passion fruit, papaya, tomato, mango, bell-pepper, potato and kiwi) containing homologous molecules without any previous sensitization to the fruit proteins [125]. Patients with fruit allergy, but without symptoms against latex, can have latex specific IgE recognizing allergens of *Hevea brasiliensis*. Some of these patients show clinical reactions after latex challenge, but in all of them the clinical symptoms to fruits preceded a history of latex allergy [126].

OAS is one of the most common symptoms in latex-fruit related allergy even if some patients can also suffer from contact urticaria, angioedema, conjunctivitis, generalized urticaria and anaphylaxis. The main allergenic proteins reported to be responsible for this syndrome are: Hev b 2 (β-1,3-glucanase), Hev b 5-like proteins, Hev b 7 (patatin-like protein), Hev b 6 (hevein-like domain), Hev b 8 (profilin) and Hev b 14 (chitinase).

β-1,3-Glucanases are proteins of the PR-2 family, with a molecular mass of around 20–23 kDa. These proteins are involved in the response to pathogenic attacks and in several physiological and developmental processes [127]. This enzyme is recognized by some latex-allergic patients and it has been associated with a sensitization to bell pepper (Cap a glucanase) [128], olea pollen **(**Ole e 9) and ash pollen (Fra e 9). The clinical relevance of this cross-reactivity is still to be determined [129].

Chitinases are glycosyl hydrolases capable of the degradation of chitin, a structural component of the exoskeleton of insects and the cell walls of most fungi. They are widely distributed in plants and play an important role in the defense against chitin-containing pathogens such as fungi, nematodes and arthropods. Chitinases are classified into five classes, namely I, II, III, IV and V [130]. Class I and IV chitinases have an N-terminal hevein-like domain (HLD) that is a chitin-binding module.

Class I chitinases are present in high concentrations in some fruits (e.g. chestnut, avocado and banana) and their allergenic activity is inactivated by heat while enhanced by the artificial ripening of fruits [131]. Hev b 6 has sequence identities of more than 50% with the hevein domains of class I chitinases from fruits. Class I chitinases have often been suggested to be the main elicitor of latex-associated plant food allergy due to their cross-reactivity with hevein, the major latex allergen [132]. However, despite the high cross reactivity between these molecules, no correlation to the incidence of latex associated plant food allergies has been found [133].

The chitinase III of pomegranate, Pun g 14, is a 29-kDa protein showing a 69% sequence identity with the latex hevamine, Hev b 14, and an IgE binding in dot blotting, immunoblotting and the FABER test [23]. Ziz m 1 from Indian jujube and Rub i chitinase from raspberry are additional class III chitinases responsible for OAS and other allergic symptoms [134, 135].

Within a random population of 5307 patients analyzed with the FABER test [23], the chitinase showing the highest prevalence of IgE-positive subjects was kiwifruit class IV chitinase (2.4%), whereas lower values were observed for pomegranate chitinase III (1.3%) and hevein (1.2%). The lowest prevalence was registered for latex chitinase I (0.8%). Therefore, chitinase III and IV might have an important role in the allergic sensitization to plant food independently of the presence or absence of the hevein-like domain in their structure, thus subverting the concept of latex-fruit syndrome.

Hev b 5 is one of the most important allergens of Hevea latex. Recently, a Hev b 5 homologous protein has been identified as a new IgE-binding protein contained in peach and apricot. It has been named ENEA on the basis of the N-terminal amino acid sequence (E-N-E-A) of the natural molecule isolated from peach [17]. The amount of this protein was estimated to be very variable in different peach cultivars, and in different crops of the same cultivar. Hence, the amount of ENEA to which people are exposed when eating peach may be very variable. In addition to the major allergen from rubber latex, Hev b 5, ENEA has structural similarities with the food allergen Man e 5, identified in manioc tuber. Both, ENEA and Man e 5 show an IgE cross-reactivity with Hev b 5 and they can be responsible for OAS and generalized allergic reactions with and without a primary allergy to latex [17, 136].

Hev b 7 (patatin-like protein), a latex allergen of 46-kDa, has about 50% sequence identity with the potato allergen patatin (Sola t 1), a heat-labile allergen. Allergic symptoms, including OAS, have been described as a consequence of the cross-reactivity between Hev b 7 and potato patatin [137].

### Seed storage proteins

OAS has been described in patients suffering from nut and legume allergy [87]. In fact, several allergens are present in the kernels and seeds of edible fruits, the majority of them being seed storage proteins, classified according to their solubility in water (2S albumins) and salt-buffered solutions (globulins). Seed globulins or cupins are divided into three groups: germins (7S globulins), vicilins (7S globulins) and legumins (11S globulins). Seed storage proteins provide the necessary nutrients to plant seeds during sprouting. The 11S globulins, 2S albumins and the 7S vicilins are included in this group of allergenic proteins that are resistant to heat and digestion (class 1 food allergens). They are among the principal food allergens responsible for severe anaphylactic reactions. These proteins could also represent hidden allergens when a seed fragment is ingested as a result of the rupture of the seeds of fruits such as kiwifruit, orange and tomato.

### Cysteine proteases

Some cysteine proteases have been described as allergens in fruits such as kiwi (Act d 1), pineapple (Ana c 2), papaya (Cari p chymopapain and Cari p papain), fig (Fic c 2) and soy (Gly m Bd 30 K). They are genuine allergens, without any known cross reaction among them. Some of them cause OAS and sometimes laryngeal edema, and even anaphylactic shock such as Act d 1, the major kiwi allergen.

## Conclusions

In medical dictionaries, syndrome is a term indicating a characteristic combination of symptoms, without a precise reference to its causes and to the mechanism of the symptom onset. In line with this definition, OAS represents a complex of allergy symptoms localized to the mouth and throat, including itching and/or angioedema of the lips, tongue, palate, ears and throat, accompanied by stinging pain. Some allergic patients show OAS after the ingestion of specific foods. For a long time, literature reports have been describing a high prevalence of OAS following the ingestion of fresh plant foods in patients sensitized to pollen. To highlight this high prevalence, the expression PFAS was used many years ago and it is still accepted today. In the wake of PFAS many other syndromes have subsequently been reported. Often, when in a study population a reaction towards an allergy source appeared to be frequently associated with the reaction to another source, a syndrome was proposed, thus associating, for instance, fruit to fruit, fruit to pollen and latex to food. Indeed, in these cases the term syndrome appears to be referred to the allergenic source (pollen, fruit or latex), rather than to a characteristic combination of symptoms defining a canonical syndrome. Probably we should consider whether or not the term “syndrome” has always been used in an appropriate manner.

Until today, many authors have believed that, in pollen allergic patients, a convincing clinical history of symptoms following the ingestion of cross-reacting foods associated with positive IgE tests to the relevant allergens, is sufficient to guarantee the diagnosis of OAS. Additionally, many studies have been restricted to small groups of patients living in confined geographical locations and often selected on the basis of predefined criteria thereby probably creating some bias. A small selection of plant food allergens has been thoroughly investigated while not every allergen family has been equally considered, some being simply ignored, thus restricting the possibility of identifying subgroups of association patterns. Therefore, a co-sensitization to additional allergens could be much more common than previously believed. All these considerations suggest that there is a serious risk of biased findings. The classification into class 1 and 2 of the food allergens underlying OAS and the so-called PFAS is a good example of how great is the human need to create classifications in an attempt to distinguish phenomena that are only apparently different. Recent data emerging from literature seem to overturn these concepts by hypothesizing that pollen allergenic proteins might be capable of triggering serious food reactions only in subgroups of patients. To either confirm or deny what we have so far believed we should conduct immunological studies based on different and extensive groups of populations in which all known and yet undiscovered allergens are taken into consideration, without stopping at the first “impression” and restricting the search to a few known allergenic proteins. Several still unknown allergenic proteins could be contained in foods. These allergenic sources, while containing homologous proteins, are not always recognized by every allergic patient. The fact that so many different syndromes have been coined, at the base of which there is the sensitization to homologues proteins, constitutes the evidence that the recognition of allergenic sources is not uniform.

### The three take home messages

1. Do not mismatch the terms: The term PFAS is not equivalent to the term OAS. OAS is one of the first symptoms of food allergy. Only the exact identification of the culprit molecules provides the individual IgE sensitization profile allowing the assessment of the risk of developing more severe generalized allergic reactions.
2. Think out of the box: Never fix the OAS diagnosis on pre-established syndromes or on the most common allergenic sources or on the most common allergenic molecules.
3. Tailor the diagnosis on the specific patient: rather than tailoring the patient on a presumed diagnosis. Always look for all the involved allergenic molecules.

## Supplementary information

**Additional file 1: Table S1.** Details on Bet v 2 homologs from different sources compared with Bet v 1.0101 (see also Fig. 2a). **Table S2.** Details on Bet v 1 homologs from different sources compared with Bet v 1.0101 (see Fig. 2b). **Table S3.** Details on a selection of Bet v 1 isoallergens and isoforms, found in birch pollen and compared with Bet v 1.0101 (see also Fig. 2c). **Table S4.** Details on GRP homologs from different sources compared with Cry j GRP (see also Fig. 2d).

## Data Availability

Data sharing not applicable to this article as no datasets were generated or analysed during the current study.

## Abbreviations

LTP: Lipid transfer protein
GRP: Gibberellin regulated protein
NSAIDs: Nonsteroidal anti-inflammatory drugs
OAS: Oral allergy syndrome
PFAS: Pollen food allergy syndrome
SPT: Skin prick test
