# Supplementary material for: Molecular approach to a patient’s tailored diagnosis of the oral allergy syndrome
Source: Clin Transl Allergy. 2020 Jun 17;10:22. doi: 10.1186/s13601-020-00329-8 (PMC7298840; doi:10.1186/s13601-020-00329-8)
Supplement: Supplementary file 1 — Additional file 1: Table S1. Details on Bet v 2 homologs from different sources compared with Bet v 1.0101 (see also Fig. 2a). Table S2. Details on Bet v 1 homologs from different sources compared with Bet v 1.0101 (see Fig. 2b). Table S3. Details on a selection of Bet v 1 isoallergens and isoforms, found in birch pollen and compared with Bet v 1.0101 (see also Fig. 2c). Table S4. Details on GRP homologs from different sources compared with Cry j GRP (see also Fig. 2d). [file 13601_2020_329_MOESM1_ESM.pdf]

Molecular approach to a patient's tailored diagnosis of the oral allergy syndrome

Alessandri Claudia[1,2], Ferrara Rosetta[1,2], Bernardi Maria Livia[1,2], Zennaro Danila[1,2], Tuppo Lisa[2,3], Giangrieco Ivana[2,3], Ricciardi Teresa[2,3], Tamburrini Maurizio[3], Ciardiello Maria Antonietta[3], Mari Adriano[1,2]

[1]Associated Centers for Molecular Allergology (CAAM), Rome, Italy.

[2]Allergy Data Laboratories (ADL), Latina, Italy

[3]Institute of Biosciences and BioResources (IBBR), CNR, Naples, Italy.

Correspondence:

Claudia Alessandri

Centri Associati di Allergologia Molecolare, Rome, Italy

Tel: +39 3337637667

email: claudia.alessandri@caam-allergy.com

Table S1. Details on Bet v 2 homologs from different sources compared with Bet v 1.0101 (see also Figure 2a)

Allergen	Accession number (Uniprot)	Plant order	Source	Tissue	Identity with Bet v 2.0101 (%)
**Bet v 2.0101**	P25816	*Fagales*	birch	pollen	100
**Cor a 2**	A4KA45	*Fagales*	hazelnut	pollen, seed	92
**Jug r 7**	A0A2I4	*Fagales*	walnut	seed	90
**Gly m 3**	I1K602	*Fabales*	soybean	seed	86
**Vit v 4**	A5BLM8	*Vitales*	grape	fruit	86
**Fra e 2**	W8NXD0	*Lamiales*	ash	pollen	85
**Ole e 2**	A4GDU2	*Lamiales*	olive tree	pollen	85
**Ole e 2**	O24169	*Lamiales*	olive tree	pollen	84
**Mer a 1.0101**	O49894	*Malpighiales*	annual mercury	pollen	84
**Jug r 7.0101**	A0A2I4DNN6	*Fagales*	walnut	seed	83
**Lil l 2**	Q9SNW7	*Liliales*	easter lily	pollen	82
**Mal d 4.0302**	Q84RR7	*Rosales*	apple	fruit	82
**Pyr c 4.0101**	Q9XF38	*Rosales*	pear	fruit	82
**Mal d 4.0301**	Q9XF40	*Rosales*	apple	fruit	81
**Dau c 4.0101**	Q8SAE6	*Apiales*	carrot	root	80
**Hev b 80201**	Q9M7N0	*Malpighiales*	rubber tree	latex	80

Table S2. Details on Bet v 1 homologs from different sources compared with Bet v 1.0101 (see Figure 2b)

Allergen	Accession number (Uniprot)	Plant Order	Source	Tissue	identity with Bet v 1.0101
**Bet v 1.0101**	P15494	*Fagales*	birch	pollen	100
**Cor a 1.0201**	Q39453	*Fagales*	hazelnut	pollen	83
**Aln g 1.0101**	P38948	*Fagales*	alder	pollen	81
**Car b 1.0302**	Q96382	*Fagales*	horbeam	pollen	78
**Que su 1**	H9NJ55	*Fagales*	oak	pollen	72
**Cas s 1**	Q93YH9	*Fagales*	chestnut	pollen	68
**Cas s 1**	Q93YH9	*Fagales*	chestnut	seed	68
**Jug r 5.0101**	A0A1J0	*Fagales*	walnut	seed	66
**Fag s 1.0101**	B7TWE6	*Fagales*	beech	pollen	65
**Mal d 1.0401**	Q43550	*Rosales*	apple	fruit	65
**Vit v 8**	D7SY82	*Vitales*	grape	fruit	62
**Pru du 1**	B6CQS6	*Rosales*	almond	seed	61
**Pru p 1**	B6CQS6	*Rosales*	peach	fruit	60
**Pru ar 1.0101**	O50001	*Rosales*	apricot	fruit	60
**Pru av 1.0101**	O24248	*Rosales*	cherry	fruit	59

Table S3. Details on a selection of Bet v 1 isoallergens and isoforms, found in birch pollen and compared with Bet v 1.0101 (see also Figure 2c)

Allergen	Accession number (Uniprot)	Source	Tissue	Identity with Bet v 1.0101 (%)
**Bet v 1.0101**	P15494	birch	pollen	100
**Bet v 1.0109**	Q96366	“	“	99
**Bet v 1.0113**	Q96370	“	“	98
**Bet v 1.0108**	Q96365	“	“	97
**Bet v 1.0119**	Q0QLS9	“	“	96
**Bet v 1.0102**	P43177	“	“	95
**Bet v 1.0103**	P43178	“	“	95
**Bet v 1.0118**	Q9SCH6	“	“	94
**Bet v 1.2001**	Q39429	“	“	90
**Bet v 1.0204**	Q39429	“	“	89
**Bet v 1.0205**	Q39427	“	“	88
**Bet v 1.0207**	Q0QLV2	“	“	88
**Bet v 1.0202**	P43176	“	“	87
**Bet v 1.0301**	Q39415	“	“	72

Table S4. Details on GRP homologs from different sources compared with Cry j GRP (see also Figure 2d)

Allergen	Accession number (Uniprot or GenBank)	Plant Order	Source	Tissue	identity with Cry j GRP
**Cry j GRP**	GenBank: BY902962.1	*Cupressales*	cedar	pollen	100
**Pru av 7.0101**	GenBank: XP_021820299.1	*Rosales*	cherry	fruit	79
**Pru m 7.0101**	GenBank:XP_016648029.1	*Rosales*	japanese apricot	fruit	78
**Pru p 7.0101**	P86888	*Rosales*	peach	fruit	78
**Pun g 7**	A0A218X6T8	*Myrtales*	pomegranate	fruit	78
**Cit s 7**	A0A067GPB8	*Sapindales*	sweet orange	fruit	75
